# MiR-27a-3p enhances the cisplatin sensitivity in hepatocellular carcinoma cells through inhibiting PI3K/Akt pathway

**DOI:** 10.1042/BSR20192007

**Published:** 2021-12-08

**Authors:** Ying Yang, Zhifang Yang, Ruili Zhang, Chunli Jia, Rui Mao, Shaya Mahati, Yuefen Zhang, Ge Wu, Yan na Sun, Xiao yan Jia, Ainiwaer Aimudula, Hua Zhang, Yongxing Bao

**Affiliations:** Department of Cancer Center, The First Affiliated Hospital of Xinjiang Medical University, Urumqi, China

**Keywords:** Cisplatin, Hepatocellular cancer, miR-27a-3p, PI3K/Akt pathway, Sensitivity

## Abstract

MicroRNAs (miRNAs) play an important role in drug resistance, and it is reported that miR-27a-3p regulated the sensitivity of cisplatin in breast cancer, lung cancer and ovarian cancer. However, the relationship between miR-27a-3p and chemosensitivity of cisplatin in hepatocellular carcinoma (HCC) was unclear, especially the underlying mechanism was unknown. In the present study, we analyzed miR-27a-3p expression levels in 372 tumor tissues and 49 adjacent tissues in HCC samples from TCGA database, and found that the miR-27a-3p was down-regulated in HCC tissues. The level of miR-27a-3p was associated with metastasis, Child–Pugh grade and race. MiR-27a-3p was regarded as a favorable prognosis indicator for HCC patients. Then, miR-27a-3p was overexpressed in HepG2 cell, and was knocked down in PLC cell. Next, we conducted a series of *in vitro* assays, including MTT, apoptosis and cell cycle assays to observe the biological changes. Further, inhibitor rate and apoptosis rate were detected with pre- and post-cisplatin treatment in HCC. The results showed that overexpression of miR-27a-3p repressed the cell viability, promoted apoptosis and increased the percentage of cells in G_0_/G_1_ phase. Importantly, overexpression of miR-27a-3p significantly increased the inhibitor rate and apoptosis rate with cisplatin intervention. Besides, we found that miR-27a-3p added cisplatin sensitivity potentially through regulating PI3K/Akt signaling pathway. Taken together, miR-27a-3p acted as a tumor suppressor gene in HCC cells, and it could be useful for modulating cisplatin sensitivity in chemotherapy.

## Introduction

Hepatocellular carcinoma (HCC) is one of the most common and highly lethal malignant tumors of digestive system worldwide [1]. The 5-year overall survival rate is merely 12% for most of the patients who are already at the advanced stage of HCC at the time of diagnosis [2]. Therefore, systematic treatments are recommended by experts in the HCC guidelines. These options including targeted drugs of Sorafenib and Lenvima, as well as systematic chemotherapy. However, the treatment effect of the chemotherapy was generally unsatisfactory [3,4]. Several cytotoxic agents, including cisplatin, doxorubicin and 5-florouracil (5-FU) have shown multiple drug resistance which limit their therapeutic efficacy [5]. Therefore, it is urgent to explore the molecular targets to improve the sensitivity of these cytotoxic drugs in HCC.

MicroRNAs (miRNAs) refer to a group of small and non-coding RNAs, which are 22 nucleotides in length. Its main function is to regulate gene expression at the translation level. Recently, it is reported that aberrant expression of miRNAs can modulate cell growth, apoptosis as well as tumorigenesis [6]. Besides, miRNAs can also make contributions to the chemosensitivity in HCC [7,8]. However, the underlying molecular mechanisms of chemosensitivity have not been clarified.

MiR-27a-3p is located on chromosome 19 (19p13.1), which is expressed in multiple malignant tumors, such as renal carcinoma, oral squamous cell carcinoma and pancreatic cancer and so on [9–12]. In addition, it is reported that miR-27a-3p plays a vital role in invasion, metastasis and epithelial–mesenchymal transition in HCC [13]. Furthermore, miR-27a-3p also regulates the sensitivity of cisplatin in breast cancer, lung cancer and ovarian cancer [14–16]. Nevertheless, the relationship between miR-27a-3p and chemosensitivity of cisplatin in HCC is unknown, and its underlying mechanism needs to be explored.

Thus, in the present study, we intend to assess the effect of miR-27a-3p in cisplatin treatment of HCC, and try to identify its mechanism. We found that miR-27a-3p is an indicator of favorable prognosis in HCC patients. Beside *in vitro* assays, up-regulation of miR-27a-3p decreased the cell viability, promoted the apoptosis and blocked cells in G_0_/G_1_ phase. Importantly, overexpression of miR-27a-3p markedly increased the inhibitor rate and apoptosis rate when cisplatin was added in HCC cells.

In contrast, knockdown of miR-27a-3p significantly showed an opposite trend. In addition, Western blot revealed that miR-27a-3p plus cisplatin revealed weaker expressions of PI3K and p-Akt and stronger level of C-caspase-3. Thus, PI3K/Akt pathway probably mediated this process. Hence, miR-27a-3p added cisplatin sensitivity potentially through regulating PI3K/Akt signaling pathway.

## Materials and methods

### TCGA data analysis

The online accessible TCGA data portal (https://tcga-data.nci.nih.gov/tcga/) was used. We mainly focused on miR-27a-3p expression and clinical data of HCC patients, including age, sex, race, TNM stage, grade and Child–Pugh stage. All values were collected and analyzed from 372 HCC patients. MiR-27a-3p expression was quantified using RSEM based on the TCGA methods. The upper quartile data were normalized according to the TCGA normalization protocol.

### Cell culture

Hepatoma cell lines (HepG2, Huh-7, PLC) and the human normal liver cell line LO2 were purchased from the Cell Bank of the Chinese Academy of Science (Shanghai, China). All cell lines were cultured in RPMI 1640 medium (Life Technologies, U.S.A.), supplemented with 10% fetal bovine serum (Life Technologies, U.S.A.) and cultured in a humidified atmosphere containing 5% CO_2_ at 37°C.

### Reagents

The miExpress™ Precursor miRNA Expression (Lot No. 21895-1), inhibitor expression (Lot No. B302), clone of miR-27a-3p and control were purchased from GenePharma Company (Shanghai, China). The following antibodies were used in the study: anti-PI3K, anti-Akt, anti-p-Akt, anti-C-caspase were obtained from Cell Signaling Technology (Beverly, MA). The PI3K/p-Akt signaling inhibitor LY49002 was purchased from Apicent Biological Technology Company (Shanghai, China). β-actin was purchased from Bioworld Technology (CA, U.S.A.).

### Cell transfection

Before transfection, a total of 1.5 × 10^5^ HCC cells were seeded into six-well plates for 24 h. Lipofectamine 3000 (Invitrogen, U.S.A.) was used for the transient transfection according to the manufacturer’s instructions. HepG2 cells were chosen for miR-27a-3p overexpression by plasmid transfection and PLC cells for miR-27a-3p knockdown using siRNA transient transfection. Cells were divided into four groups, miR-27a-3p overexpression and control were referred to as miR-27a and miR-Con, respectively; miR-27a-3p inhibitor and inhibitor control were named as miR-inhibitor-27a and miR-inhibitor-Con, respectively. The expression levels of miRNAs were confirmed by qRT-PCR assay.

### Cell viability and proliferation assay

Twenty-four hours after cell transfection, cell viability was identified by 3-(4,5-Dimethylthiazol-2-yl)-2-5-(3-carboxymethoxyphenyl)-2-(4-sulfophenyl)-2H-tetrazolium assay (MTT, Promega, U.S.A.). HepG2 and PLC cells were triplicate plated in a 96-well plate at the density of 5 × 10^3^ cells/well and incubated overnight. Then, the cells were treated with different concentrations of cisplatin: 0, 3, 6, 9, 12 µg/ml for 48 h, respectively. Subsequently, the MTT reagent (20 µl) was added to each well, followed by incubation at 37°C in 5% CO_2_ atmosphere for 4 h. Lastly, the absorbance was read by using a Synergy 2 (BioTek, U.S.A.) plate reader.

### Cell apoptosis and cell cycle analysis by FACS

Annexin V/propidium iodide (Av/PI) staining (Beyotime Biotechnology, China) was analyzed by flow cytometry. Cells were collected and washed twice with phosphate-buffered saline (PBS), followed by resuspension in 250 μl binding buffer. Five microliters of FITC–Annexin V and 10 μl PI (20 µg/ml) were added to each 100-μl cell suspension, and then the cells were incubated at room temperature for 15 min. Subsequently, 400 μl PBS was added to the cell suspensions, and the samples were detected by flow cytometry (Becton-Dickinson, U.S.A.). The percentage of the cells in different phases was counted and compared.

For cell cycle analysis, cells were cultured in serum-free medium for 24 h to induce cell cycle synchronization. Cells were harvested at different time points. For DNA content analysis, cells were fixed in 70% ethanol, rehydrated in PBS, treated with RNase A (10 mg/ml) for 30 min, then stained with PI (10 µg/ml) for 5 min. The percentage of cells in the S, G_0_/G_1_ and G_2_/M phases was counted and compared.

### Western blot

Cells were harvested and washed twice with PBS (HyClone, Logan, UT). Total protein was extracted using RIPA cell lysis buffer (Beyotime Biotechnology, China). Protein concentration was determined by the bicinchoninic acid protein assay (Pierce, U.S.A.), and proteins were separated by sodium dodecyl sulfate/polyacrylamide gel electrophoresis (SDS/PAGE) and blotted on to polyvinylidene difluoride (PVDF) membranes (Millipore, MA). The membranes were incubated with primary antibodies in blocking buffer overnight at 4°C. The membrane was washed three times for 5 min each time with washing buffer and incubated with secondary antibodies (Invitrogen, U.S.A.) for 1.5 h at room temperature. The proteins were visualized with the Western Breeze Kit (WB7105, Invitrogen, U.S.A.) and analyzed with Quantity One software (Bio-Rad Laboratories, U.S.A.).

### Statistical analysis

All statistical analysis was performed using SPSS software, version 17.0 (SPSS, Chicago, U.S.A.). The results are expressed as the mean ± standard deviation (SD). The data were compared among groups by one-way analysis of variance followed by Bonferroni’s correction. Each experiment was done independently at least three times. *P*-value <0.05 was considered as statistically significant difference.

## Results

### MiR-27a-3p was down-regulated in tumor tissue in HCC patients

**Figure 1:**
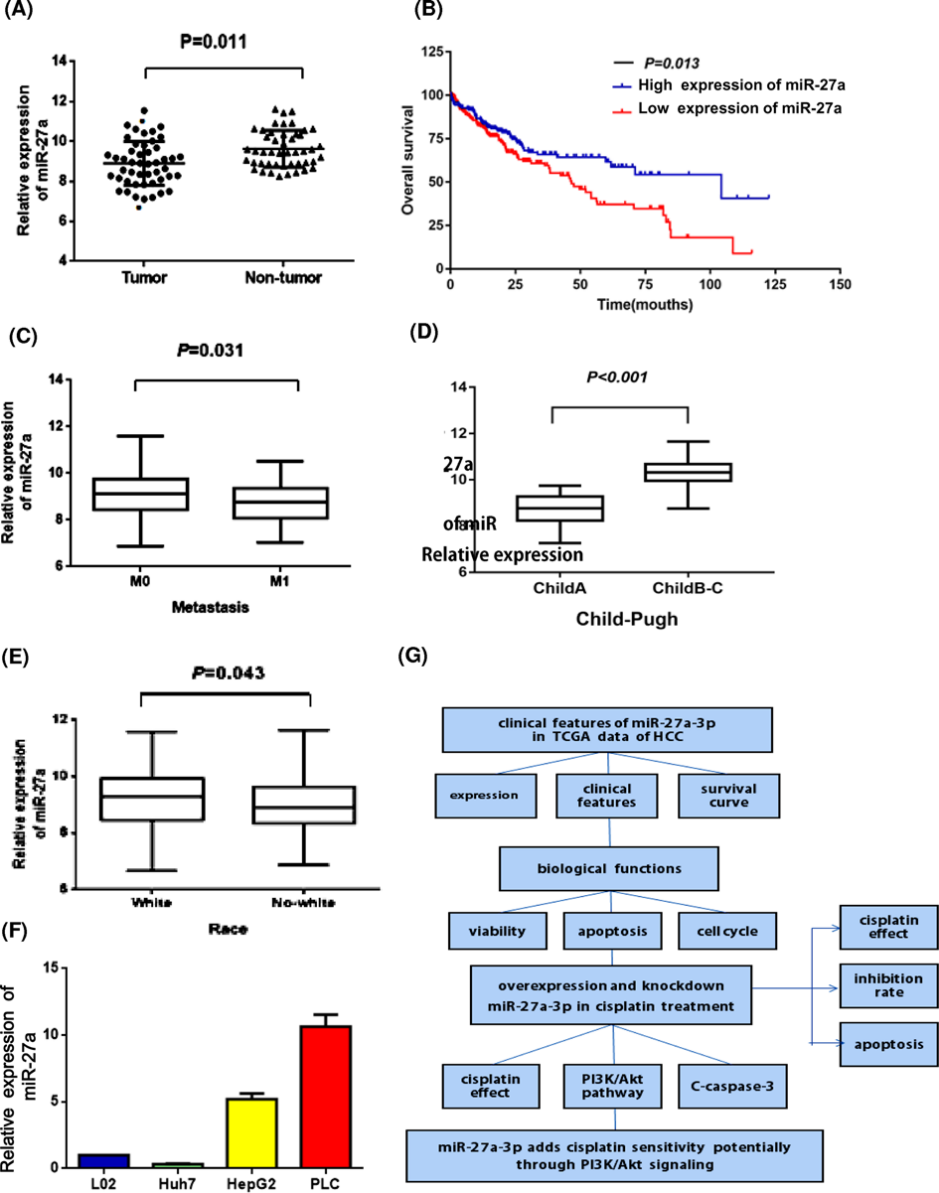
MiR-27a-3p was down-regulated in tumor tissue in HCC patients (**A**) The scatter diagram represented the expression of miR-27a-3p in 49 paired HCC tissues and adjacent non-tumor tissues, which showed that miR-27a-3p was significantly low-expressed in tumor tissue. (**B**) The Kaplan–Meier curve revealed that high level of miR-27a-3p significantly correlated with better overall survival in HCC patients. (**C–E**) The correlation analysis reflected that miR-27a-3p expressions were associated with metastasis, Child–Pugh grade and race in HCC patients. (**F**) The miR-27a-3p expressions in different cell lines were measured by RT-qPCR. (**G)**The research flow chart of the article.

To explore the clinical significance of miR-27a-3p in HCC patients, we downloaded the clinical data and miR-27a-3p expression in TCGA dataset. A total of 372 tumor tissues and 49 adjacent normal tissues were involved. Results showed that miR-27a-3p was significantly low-expressed in tumor tissue (Figure 1A). And Kaplan–Meier survival curve revealed that high level of miR-27a-3p significantly correlated with better overall survival in HCC patients (Figure 1B). Moreover, miR-27a-3p expression was associated with metastasis, Child–Pugh grade and race (Figure 1C–E), but not with the T stage, N stage and differentiation grade. The correlation between miR-27a-3p level and clinicopathological features in HCC patients is shown in Tables 1 and 2. Taken together, these results reflected that miR-27a-3p level was an indicator for favorable prognosis of HCC patients.

**Table 1:** The correlation between clinicopathological features and miR-27a-3p expression in HCC patients

Clinical characteristics	Expression				χ^2^	*P*-value
	Low		High			
	186	50.00%	186	50.00%		
Age (years)						
≤60	86	23.10%	91	24.50%	0.269	0.604
>60	100	26.90%	95	25.50%		
Gender						
Female	56	15.10%	63	16.90%	0.605	0.437
Male	130	34.90%	123	33.10%		
Race						
White	79	21.20%	103	27.70%	6.196	0.013^*^
No-white	107	28.80%	83	22.30%		
T stage						
T1–2	147	39.50%	132	35.50%	3.226	0.072
T3–4	39	10.50%	54	14.50%		
N stage						
N0	161	43.30%	170	45.70%	2.220	0.136
N1	25	6.70%	16	4.30%		
M stage						
M0	159	42.70%	172	46.20%	4.633	0.031^*^
M1	27	7.30%	14	3.80%		
Differentiation grade						
G1–2	117	31.50%	118	31.70%	0.012	0.914
G3–4	69	18.50%	68	18.30%		
Child–Pugh						
Grade A	134	36.00%	160	43.00%	10.966	0.001^*^
Grade B–C	52	14.00%	26	7.00%		
Tumor stage						
I–II	113	30.40%	112	30.10%	0.011	0.916
III–IV	73	19.60%	74	19.90%		

* *P*<0.05.

**Table 2:** Univariate and multivariate analyses of overall survival in patients with HCC

Covariate	Univariate analysis		Multivariate analysis			
	χ^2^	*P*-value	HR	(95% CI)		*P*-value
Age	1.600	0.206	1.321	0.384	3.989	0.721
Gender	1.165	0.280	1.645	0.381	5.542	0.285
Race	0.990	0.320	1.725	0.426	5.892	0.361
AFP	0.093	0.761	0.895	0.325	2.462	0.829
Cirrhosis	1.080	0.299	1.895	0.578	6.200	0. 291
Tumor size	28.902	<0.001^*^	0.135	0.042	0.052	<0.001^*^
Lymph node metastasis	0.163	0.686	0.285	0.426	1.892	0.648
Distant metastasis	4.031	0.045^*^	0.047	0.232	0.067	0.011^*^
Differentiation grade	0.192	0.661	0.296	0.431	1.910	0.653
Child–Pugh Grade	8.018	0.005^*^	1.103	0.061	0.461	0.015^*^
Tumor stage	24.084	<0.001^*^	0.723	0.057	1.201	0.061
miR-27a-3p	6.088	0.014^*^	1.117	0.013	0.291	0.010^*^

* *P*<0.05.

### MiR-27a-3p acted as a tumor suppressor gene in HCC

As miR-27a-3p was low-expressed in HCC tumor tissue, we speculated that miR-27a-3p may function as a tumor suppressor gene. Thus, we conducted a series of *in vitro* assays to explore its biological function. First, we detected the expression of miR-27a-3p in several hepatoma cell lines (HepG2, Huh-7 and PLC) and one normal liver cell line (L02). RT-qPCR results showed that miR-27a-3p expressions differed in these cells. HepG2 cells had a relatively low level whereas PLC cells had a relatively high expression (Figure 1F). Therefore, HepG2 cells were chosen for miR-27a-3p overexpression and PLC cells for miR-27a-3p knockdown.

**Figure 2:**
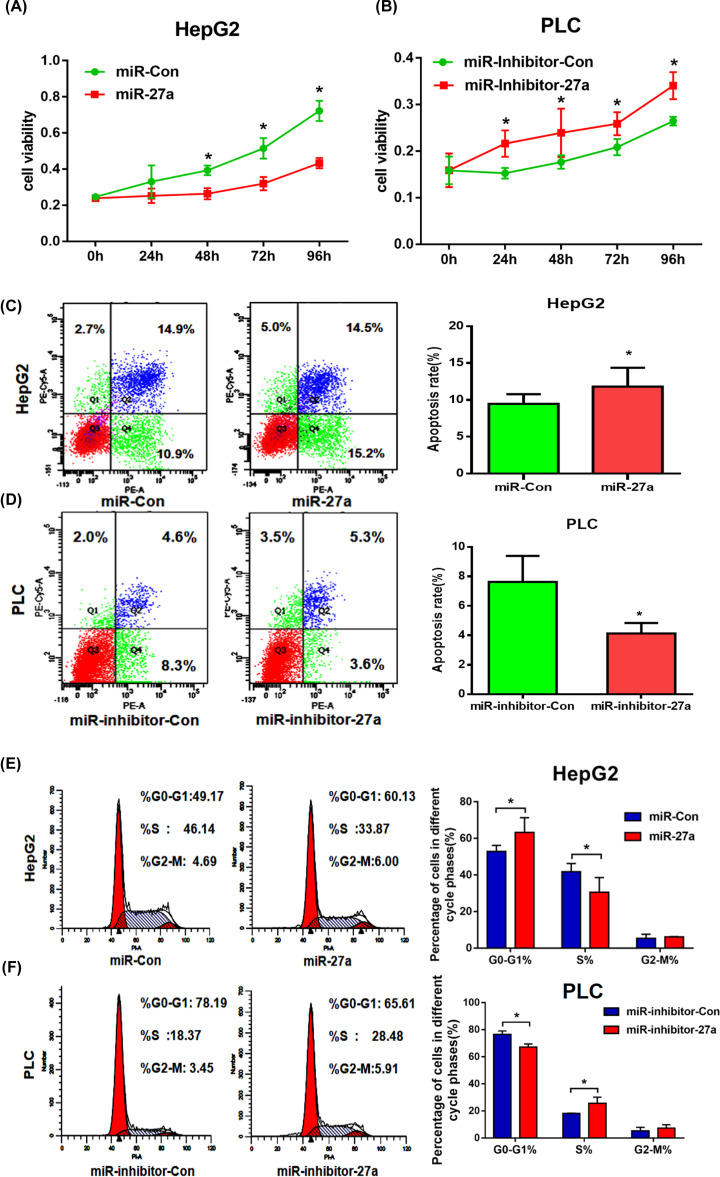
MiR-27a-3p acted as a tumor suppressor gene in HCC HepG2 cells were chosen for miR-27a-3p overexpression and PLC cells were selected for miR-27a-3p knockdown. (**A**,**B**) MTT assays showed that high level of miR-27a-3p impaired the cell viability in HepG2, whereas its low level added viability in PLC. (**C**,**D**) Up-regulation of miR-27a-3p increased the apoptosis rate in HepG2. In contrast, knockdown of miR-27a-3p significantly reduced the apoptosis rate in PLC. (**E**,**F**) The cell cycle assays revealed that overexpression of miR-27a-3p led to an increase in G_0_/G_1_ phase and a decrease in S-phase in HepG2, while its knockdown resulted in a reverse trend in PLC. All experiments were performed in triplicate. *P<0.05.

Second, MTT assay showed that high expression of miR-27a-3p impaired the cell viability in HepG2, whereas low level of miR-27a-3p added viability in PLC (Figure 2A,B). Besides, flow cytometry showed that overexpression of miR-27a-3p increased the apoptosis rate compared with miR-Con group in HepG2. In contrast, knockdown of miR-27a-3p significantly reduced the apoptosis rate in PLC (Figure 2C,D). In addition, cell cycle analysis indicated that the overexpression of miR-27a-3p led to an increase in G0/G1 phase and a decrease in S-phase in HepG2, while knockdown of miR-27a-3p resulted in a reverse trend in PLC (Figure 2E,F).

For miR-27a-3p has the function of inhibiting cell viability, inducing cell apoptosis, as well as affecting the cell cycle progression, it suggests that miR-27a-3p plays a key role in tumor suppression in the development of HCC.

### MiR-27a-3p enhanced the cisplatin sensitivity of HCC cells

**Figure 3:**
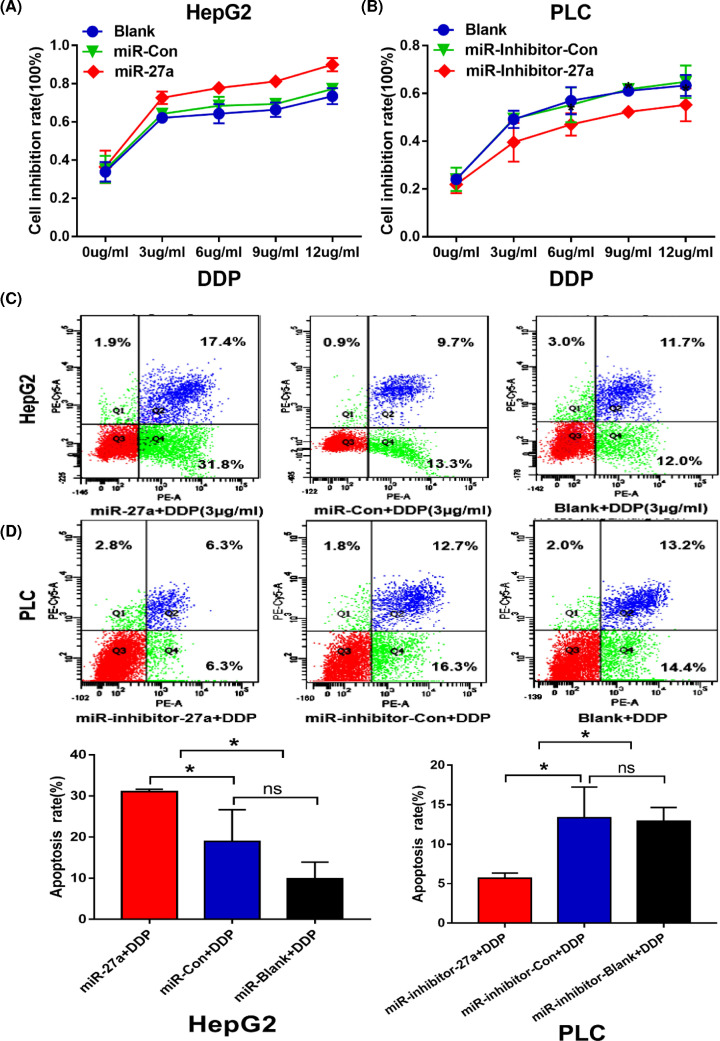
MiR-27a-3p enhanced the cisplatin sensitivity of HCC cells (**A**,**B**) MTT assays showed that overexpression of miR-27a-3p increased the inhibition rate in HepG2. On the contrary, knockdown of miR-27a-3p significantly decreased the inhibition rate in PLC. (**C**,**D**) MiR-27a+DDP group had higher apoptosis rate than that in miR-Con+DDP group in HepG2 (*P<0.05). MiR-inhibitor-27a+DDP group had lower apoptosis rate than that in miR-inhibitor-Con+DDP group in PLC (*P<0.05).

To explore whether miR-27a-3p could affect the chemosensitivity of cisplatin in HCC, we treated HepG2 and PLC cells with different concentrations of cisplatin (0, 3, 6, 9 and 12 µg/ml) and examined the effects of miR-27a-3p on cisplatin treatment. Firstly, we observed that the cell inhibition rates were gradually increased with the elevated concentration of cisplatin in blank group in both HepG2 and PLC. It indicated that the killing effect of cisplatin (Figure 3A,B). Interestingly, with cisplatin stimulation, MTT assay showed that overexpression of miR-27a-3p had the higher inhibition rate than that in miR-Con group in HepG2. On the contrary, knockdown of miR-27a-3p significantly decreased the inhibition rate compared with miR-inhibitor-Con group in PLC (Figure 3A,B).

Subsequently, we selected a moderate concentration of cisplatin (3 µg/ml DDP) for further intervention. As revealed in Figure 3C,D, cisplatin had the effects of inducing apoptosis in blank+DDP group in both HepG2 and PLC. When miR-27a-3p was overexpressed, the apoptosis rate in miR-27a+DDP group (31.8%) was higher than miR-Con+DDP group (13.3%, *P*<0.05). When miR-27a-3p was knocked down, the apoptosis rate in miR-inhibitor-27a+DDP group (6.3%) was lower than miR-inhibitor-Con+DDP group (16.3%, *P*<0.05). Collectively, we found that more inhibition rate and more apoptosis rate exhibited when miR-27a-3p was overexpressed in treatment of cisplatin in HCC cells. Thus, it implies that high level of miR-27a-3p obviously enhances cisplatin sensitivity in HCC cells.

### MiR-27a-3p added the cisplatin sensitivity potentially by inhibiting PI3K/Akt pathway

**Figure 4:**
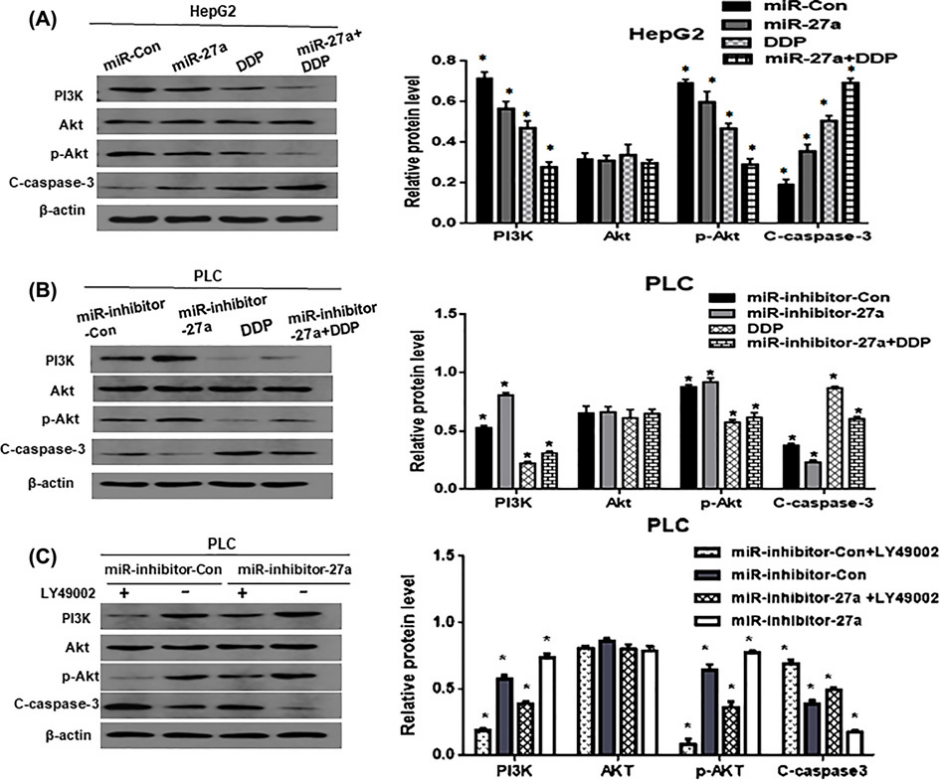
MiR-27a-3p added the cisplatin sensitivity potentially by inhibiting PI3K/Akt pathway (**A**,**B**) MiR-27a group inhibited PI3K/p-Akt signaling and elevated C-caspase-3 in HepG2. MiR-inhibitor-27a group exhibited the opposite trend in PLC cells. And DDP group showed suppression of PI3K/p-Akt pathway and up-regulation of C-caspase-3. MiR-27a+DDP group showed more obvious trend than DDP group in HepG2. MiR-inhibitor-27a+DDP group revealed stronger expression in PI3K protein and weaker level of C-caspase-3, compared with DDP group in PLC. Silencing of miR-27a-3p attenuated the cisplatin effect. (**C**) The PI3K/Akt pathway inhibitors LY49002 was used, a significant suppression of PI3K/Akt signaling and an increased expression of C-caspase-3 was observed. When miR-27a-3p was knocked down, the above trend was attenuated in miR-inhibitor-27a group, compared with miR-inhibitor-Con group. β-actin was used as a loading control. All of the experiments were performed in triplicate. *P<0.05.

In order to show the potential mechanism by which miR-27a-3p exerted anti-cancer effects in HCC cells, the signaling pathway proteins were assessed. Firstly, as revealed in Figure 4A, compared with miR-Con group, overexpression of miR-27a-3p inhibited PI3K/p-Akt signaling and elevated C-caspase-3 in HepG2. Nevertheless, PLC cells exhibited an opposite trend, compared with miR-inhibitor-Con group, silencing of miR-27a-3p activated the PI3K/p-Akt signaling and resulted in reduced C-caspase-3 level (Figure 4B). In addition, it is known that cisplatin has anti-cancer effect. And DDP group exhibited the suppression of PI3K/p-Akt pathway and up-regulation of C-caspase-3. Interestingly, miR-27a+DDP group showed the identical effect, and which was more obvious than that in DDP group in HepG2. It indicated that overexpression of miR-27a-3p facilitated the anti-cancer effect of cisplatin. However, the miR-inhibitor-27a+DDP group revealed stronger expressions of PI3K and p-Akt protein and weaker level of C-caspase-3, compared with DDP group in PLC. Silencing of miR-27a-3p attenuated the cisplatin effect.

With LY49002 intervention, compared with miR-inhibitor-Con group,miR-inhibitor-27a group showed only a slightly weakened expression of C-caspase-3, the effect of knockdown miR-27a-3p was attenuated (Figure 4C). This suggests that PI3K/Akt signaling is the main pathway affecting cisplatin sensitivity. And miR-27a-3p adds its sensitivity potentially through the inhibition of PI3K/Akt signaling.

## Discussion

In the present study, clinically, we mainly found that miR-27a-3p was down-regulated in HCC tissue and could be regarded as an indicator for favorable prognosis of HCC patients. And in *in vitro* assays, consistent with current studies, we noticed that miR-27a-3p acted as a tumor suppressor gene in HCC. For it had the functions of inhibiting cell viability, inducing the apoptosis and affecting the cell cycle. However, whether miR-27a-3p contributed to the sensitivity of cisplatin was unclear. Importantly, for the first time, we reported that overexpression of miR-27a-3p significantly increased the inhibition rate and apoptosis rate with cisplatin treatment in HCC cells. It suggests that miR-27a-3p play a vital role in regulating cisplatin sensitivity. Besides, we preliminarily found that miR-27a-3p added cisplatin sensitivity potentially through regulating PI3K/Akt signaling pathway. Our data highlight a novel molecular target for chemotherapy of HCC.

In clinical samples, the TCGA analysis showed that miR-27a-3p was down-regulated in HCC tissues, and was associated with metastasis, Child–Pugh Grade and race. HCC patients with a high level of miR-27a-3p had better prognosis than those with a low level. This was consistent with Zhao et al.’s findings [13]. He detected the level of miR-27a by qRT-PCR in 42 cases of HCC tissues and 35 cases of adjacent tissues. Identically, they also found that miR-27a was significantly lower in HCC tissues and it could be considered as an indicator for favorite prognosis in HCC patients.

In *in vitro* assays, we firstly verified its basic biological function in HCC cells. We demonstrated that overexpression of miR-27a-3p inhibited cell viability, promoted apoptosis and blocked the cell cycle in G_0_/G_1_ phase. It means that miR-27a-3p plays a key role in tumorigenesis of HCC. However, in other types of cancer, miR-27a-3p exerted different functions. In gastric cancer, Liu et al. reported that miR-27a worked as an oncogene of promoting cell growth [17]. And in nasopharyngeal carcinoma, Li et al. found that miR-27a-3p had the function of promoting cell proliferation, facilitating migration and invasion by targeting Mapk10 protein [18]. Besides, Mertens-Talcott et al. observed that the oncogenic role of miR-27a, which increased the percentage of breast cancer cells in G_2_-M phase by inducing target gene *Myt-1* [19]. The above evidence reflects that miR-27a-3p plays different roles in different types of cancer.

Current researches have reported that some miRNAs could regulate the sensitivity of chemotherapy drug cisplatin in multiple types of cancer, including HCC. For example, Let-7 affected the sensitivity of cisplatin by IL-6/STAT3 pathway in esophageal cancer [20]. And miR-214 led to the cell survival and cisplatin resistance by targeting PTEN in ovarian cancer [21]. Furthermore, Li et al. observed that up-regulation of miR-27a contributed to the chemoresistance of cisplatin by suppressing RKIP expression in lung cancer cells [15]. And in HCC, some studies have confirmed that several specific miRNAs affect the chemosensitivity of cisplatin in HCC, which includes miR-33a, miR-34a-5p, miR-133a, miR-193b and so on [22–25]. However, the relationship between miR-27a-3p and cisplatin sensitivity in HCC was unknown.

Thus, we aimed to explore whether miR-27a-3p could affect the sensitivity of cisplatin. We treated HCC cells with different concentrations of cisplatin. An interesting phenomenon emerged, the cell viability was obviously inhibited and the apoptosis rate was significantly added when miR-27a-3p was overexpressed with cisplatin intervention in HepG2. Yet, the reverse trend was noticed in PLC cells when miR-27a-3p was knocked down. Collectively, it proved that high level of miR-27a-3p sensitized the effect of cisplatin by suppressing cell viability and inducing cell apoptosis. As we know that multidrug resistance (MDR) is the main problem that limits the therapeutic efficiency of chemotherapy drug. And P-gp protein and its encoded gene *MDR1* have been verified to play a vital role in drug resistance. Li et al. found that miR-27a may be involved in drug resistance by regulating MDR1/P-gp protein expression in ovarian cancer cells [16].

It is reported that a variety of extracellular stimuli such as insulin and chemotherapy drugs can activate signal pathways, apart from the Smad-dependent signaling, Smad-independent signaling, including the PI3K/Akt, JAK/STAT3 and MAPK pathways [26]. These pathways exert their functions by signal cascade amplification [27]. And among them, PI3K/Akt signaling plays a critical role in affecting the sensitivity of chemotherapy drug [28]. The apoptosis rate detection is one of important methods to determine the drug sensitivity. The apoptosis pathway is mainly mediated by the caspase family protein, which determines whether the cell continues to survive or die through the internal or mitochondrial apoptosis pathway [29].

We all know that cisplatin has anti-cancer effect. In our study, DDP resulted in down-regulation of PI3K, p-Akt proteins and up-regulation of C-caspase-3 level. Notably, miR-27a+DDP showed more obvious trend than that in DDP alone, it implies that cisplatin inhibits the PI3K/Akt signaling and induces cell apoptosis, and miR-27a-3p increases the cytotoxicity of cisplatin. In contrast, when miR-27a-3p was knocked down, it showed the activation of PI3K/Akt signaling and suppression of C-caspase-3. It indicates that silencing of miR-27a-3p attenuates the cisplatin effect by affecting PI3K/Akt signaling.

To negatively validate whether miR-27a-3p affects cisplatin sensitivity by PI3K/Akt signaling, we used the pathway inhibitors LY49002. As shown in Figure 4C, when LY49002 was added, it caused a significant suppression of the PI3K/AKT signaling and an obvious increased expression of C-caspase-3. This suggests that PI3K/Akt signaling is the main pathway in increasing cisplatin sensitivity. Whereas knockdown of miR-27a-3p, the above trend was attenuated in miR-inhibitor-27a group, compared with miR-inhibitor-Con group. Thus, we infer that miR-27a-3p adds cisplatin sensitivity potentially through regulating PI3K/Akt signaling. This is supported by Zhu et al.’s results [30], he found that miR-27a-3p might be associated with resistance of breast cancer cells to adriamycin treatments, by targeting BTG2 and promoting the PI3K/Akt pathway in breast cancer cells. And the shortcoming of the present paper is that we have not determined the target gene of miR-27a-3p that involved in cisplatin intervention, and we will continue to explore in future study.

In conclusion, miR-27a-3p acts as a tumor suppressor gene in HCC. Importantly, overexpression of miR-27a-3p adds cisplatin sensitivity potentially through PI3K/Akt signaling in hepatoma cell lines. Our data highlight a molecular target in chemotherapy strategy of HCC.

## Supplementary Material

Supplementary files

## Data Availability

All data generated or analyzed during the present study are included in this published article and its Supplementary files.

## Abbreviations

HCC: hepatocellular carcinoma
MDR: multidrug resistance
miRNA: microRNA
MTT: 3-(4,5-Dimethylthiazol-2-yl)-2-5-(3-carboxymethoxyphenyl)-2-(4-sulfophenyl)-2H-tetrazolium assay
PBS: phosphate-buffered saline
PI: propidium iodide
TCGA: the cancer genome atlas
TNM: tumor node metastasis
