# Supplementary material for: MiR-27a-3p enhances the cisplatin sensitivity in hepatocellular carcinoma cells through inhibiting PI3K/Akt pathway
Source: Biosci Rep. 2021 Dec 8;41(12):BSR20192007. doi: 10.1042/BSR20192007 (PMC8661504; doi:10.1042/BSR20192007)
Supplement: Supplementary files [file BSR-2019-2007_supp.zip › BSR-2019-2007_supp.pdf]

FACSDiva Version 6.1.3

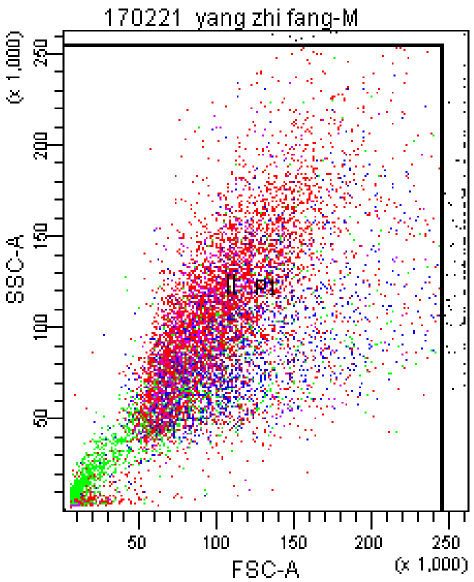

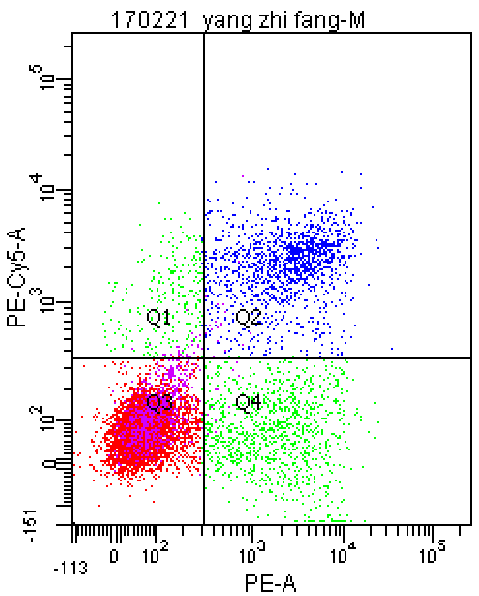

Tube: M

Population	#Events	%Parent	%Total
All Events	10,000	####	100.0
P1	9,827	98.3	98.3
Q1	269	2.7	2.7
Q2	1,462	14.9	14.6
Q3	7,025	71.5	70.2
Q4	1,071	10.9	10.7
P2	718	7.3	7.2

FACSDiva Version 6.1.3

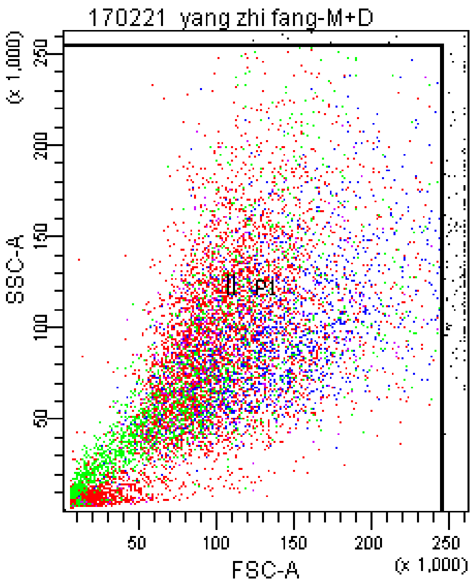

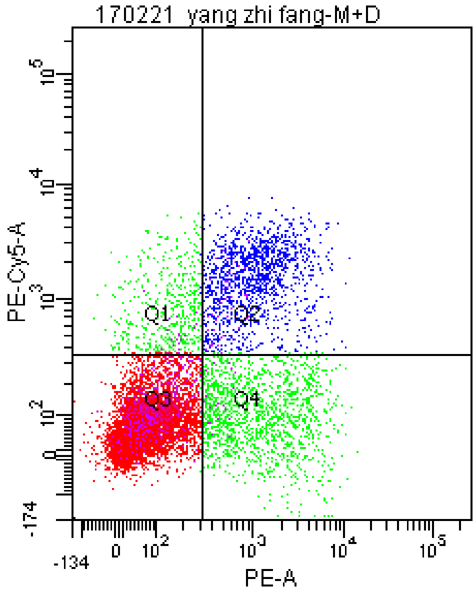

Tube: M+D

Population	#Events	%Parent	%Total
All Events	10,000	####	100.0
P1	9,775	97.8	97.8
Q1	486	5.0	4.9
Q2	1,415	14.5	14.1
Q3	6,389	65.4	63.9
Q4	1,485	15.2	14.8
P2	450	4.6	4.5

FACSDiva Version 6.1.3

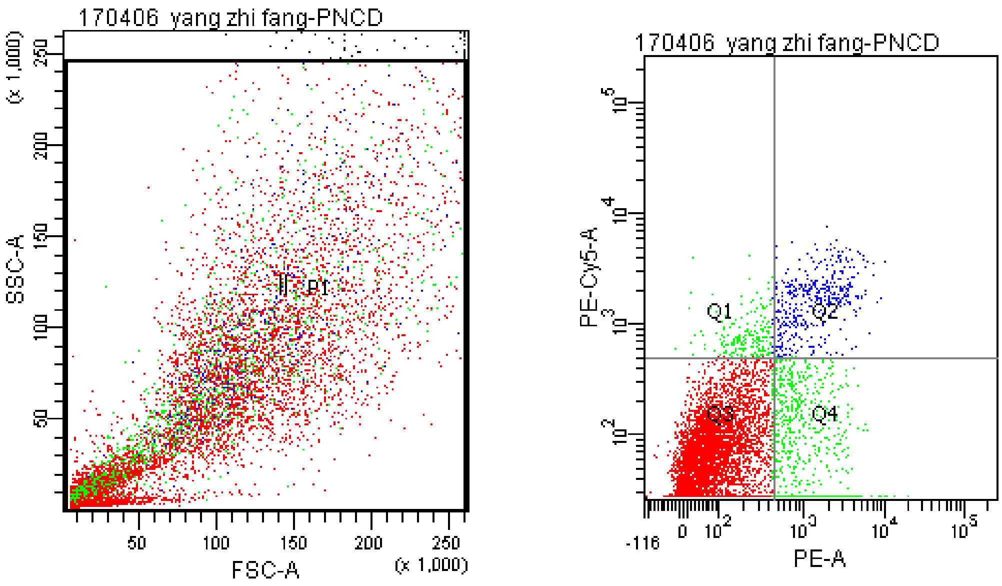

Tube: PNCD

Population	#Events	%Parent	%Total
All Events	10,000	####	100.0
P1	9,628	96.3	96.3
Q1	193	2.0	1.9
Q2	441	4.6	4.4
Q3	8,199	85.2	82.0
Q4	795	8.3	8.0

FACSDiva Version 6.1.3

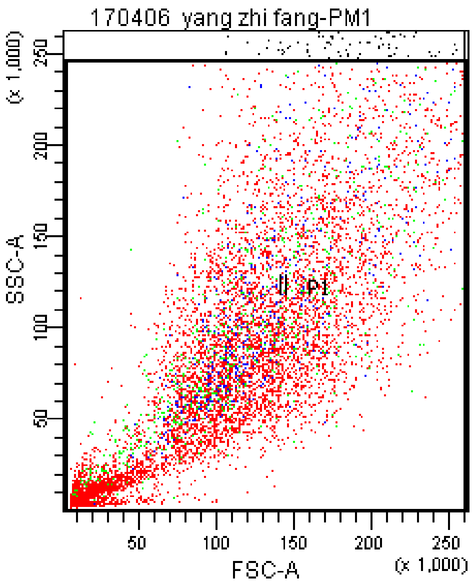

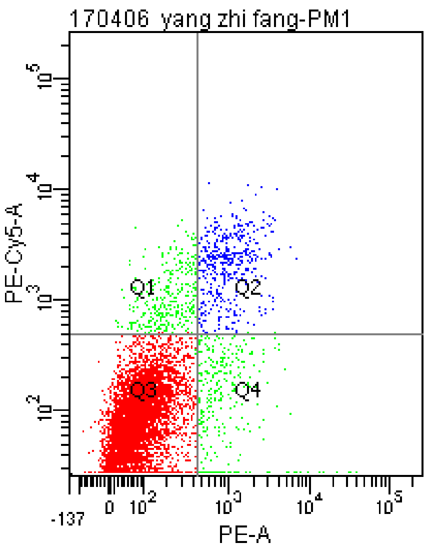

Tube: PM1

Population	#Events	%Parent	%Total
All Events	10,000	####	100.0
P1	9,078	90.8	90.8
Q1	321	3.5	3.2
Q2	483	5.3	4.8
Q3	7,948	87.6	79.5
Q4	326	3.6	3.3

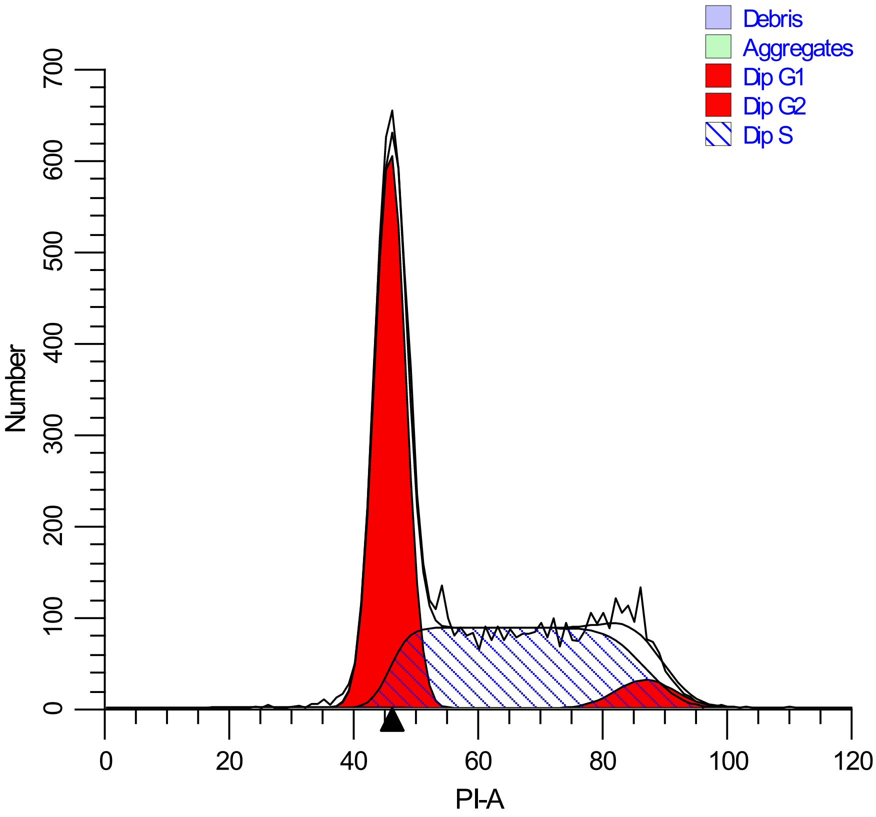

File analyzed: yang zhi fang 20170311_Tube_
Date analyzed: 11-Mar-2017
Model: 1DA0n_DSD
Analysis type: Manual analysis
Auto Linearity: No

Ploidy Mode: First cycle is diploid

Diploid: 100.00 %
Dip G1: 49.17 % at 45.65
Dip G2: 4.69 % at 86.74
Dip S: 46.14 % G2/G1: 1.90
%CV: 5.45

Total S-Phase: 46.14 %
Total B.A.D.: 0.06 %

Debris: 0.47 %
Aggregates: 0.00 %
Modeled events: 7843
All cycle events: 7806
Cycle events per channel: 185
RCS: 2.083

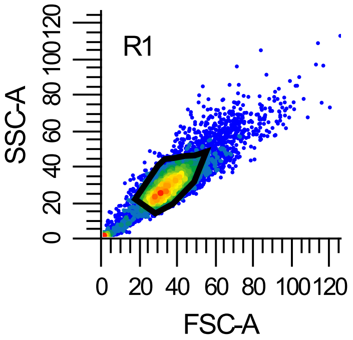

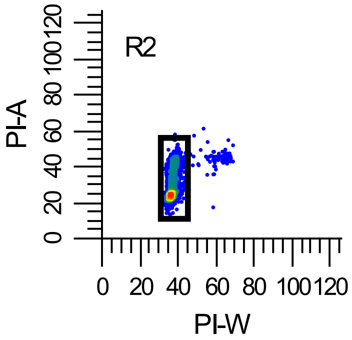

ModFit LT V4.1.7(Win)

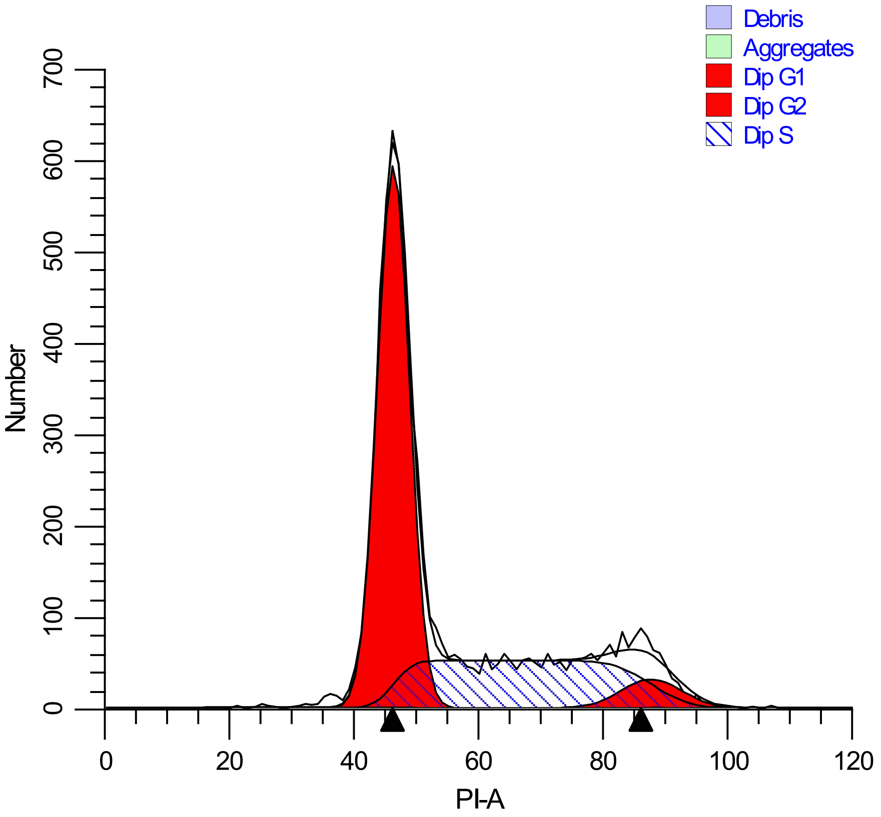

File analyzed: yang zhi fang 20170311_Tube_
Date analyzed: 11-Mar-2017
Model: 1DA0n_DSD
Analysis type: Manual analysis
Auto Linearity: No

Ploidy Mode: First cycle is diploid

Diploid: 100.00 %
Dip G1: 60.13 % at 46.15
Dip G2: 6.00 % at 87.68
Dip S: 33.87 % G2/G1: 1.90
%CV: 5.55

Total S-Phase: 33.87 %
Total B.A.D.: 0.09 %

Debris: 0.58 %
Aggregates: 0.11 %
Modeled events: 6430
All cycle events: 6386
Cycle events per channel: 150
RCS: 1.355

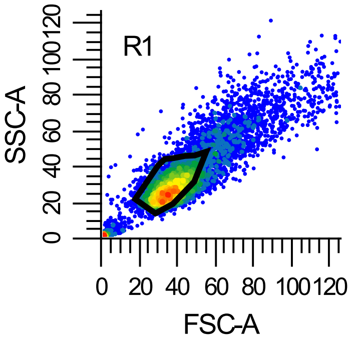

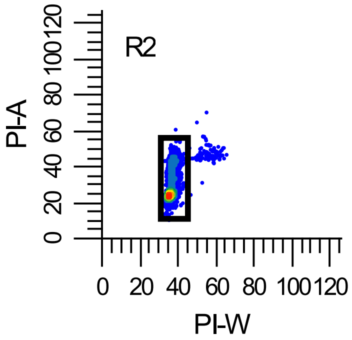

ModFit LT V4.1.7(Win)

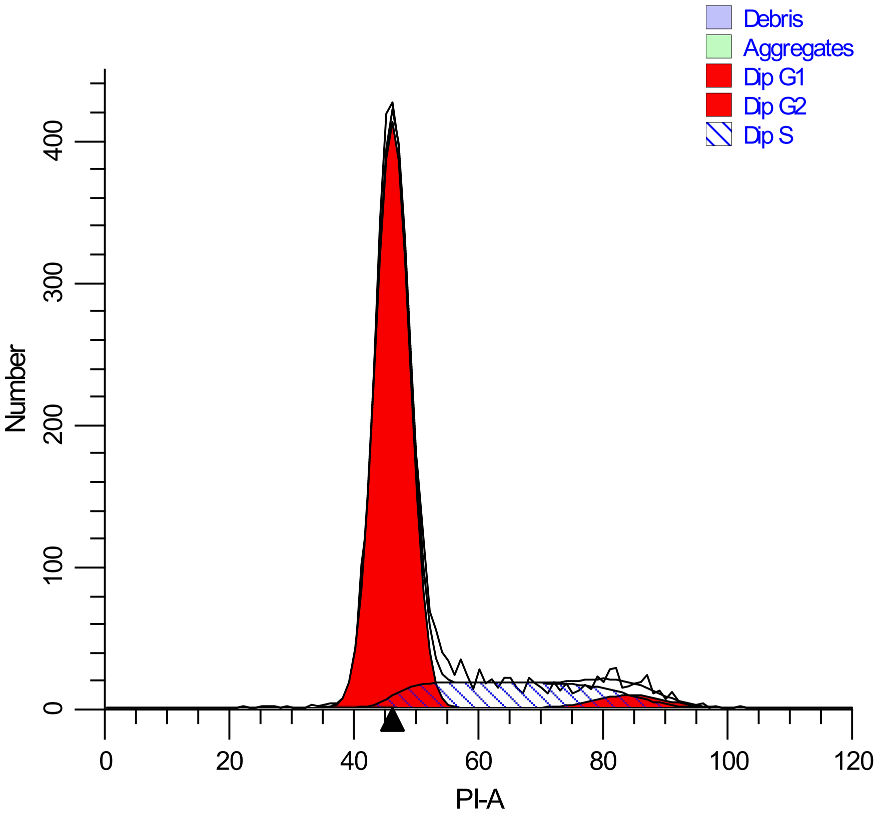

File analyzed: yang zhi fang 20170317_6_00[illegible]
Date analyzed: 17-Mar-2017
Model: 1DA0n_DSF
Analysis type: Manual analysis
Auto Linearity: No

Ploidy Mode: First cycle is diploid

Diploid: 100.00 %
Dip G1: 78.19 % at 45.99
Dip G2: 3.45 % at 84.35
Dip S: 18.37 % G2/G1: 1.83
%CV: 6.02

Total S-Phase: 18.37 %
Total B.A.D.: 0.07 %

Debris: 0.50 %
Aggregates: 0.04 %
Modeled events: 3701
All cycle events: 3681
Cycle events per channel: 94
RCS: 1.465

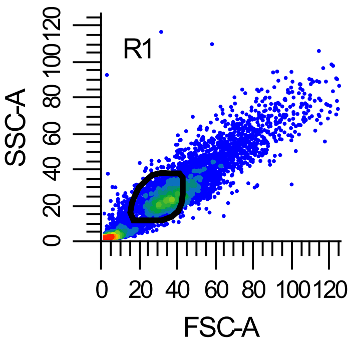

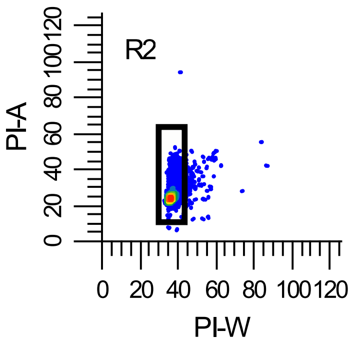

ModFit LT V4.1.7(Win)

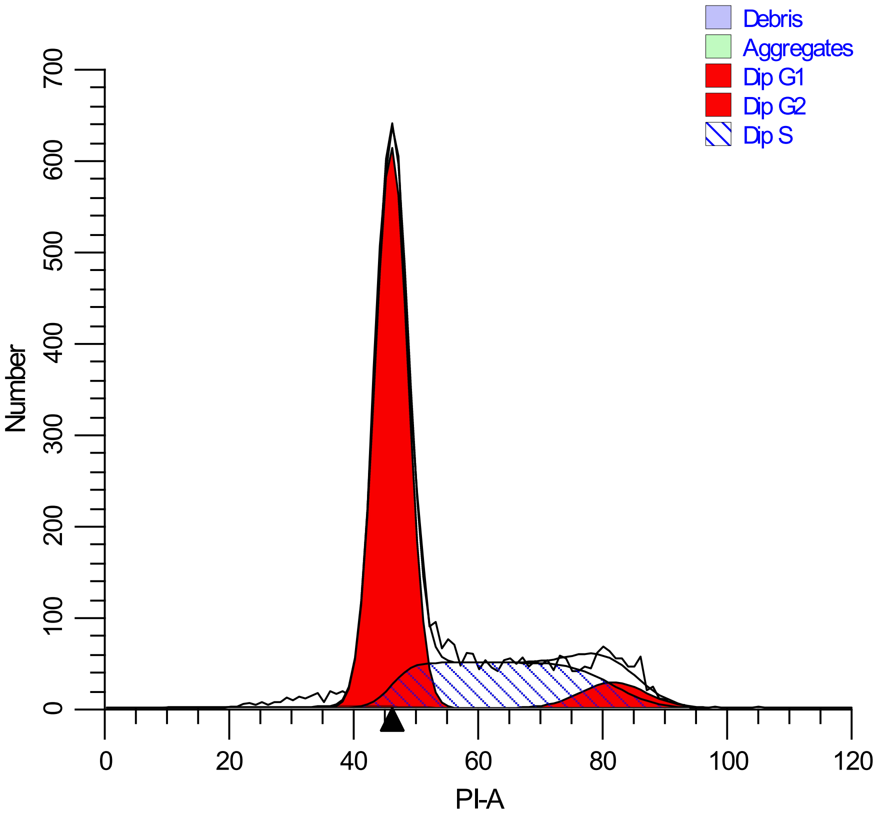

File analyzed: yang zhi fang 20170317_3_00
Date analyzed: 17-Mar-2017
Model: 1DA0n_DSF
Analysis type: Manual analysis
Auto Linearity: No

Ploidy Mode: First cycle is diploid

Diploid: 100.00 %
Dip G1: 65.61 % at 45.88
Dip G2: 5.91 % at 81.59
Dip S: 28.48 %   G2/G1: 1.78
%CV: 5.74

Total S-Phase: 28.48 %
Total B.A.D.: 0.21 %

Debris: 1.03 %
Aggregates: 0.00 %
Modeled events: 6288
All cycle events: 6224
Cycle events per channel: 170
RCS: 2.136

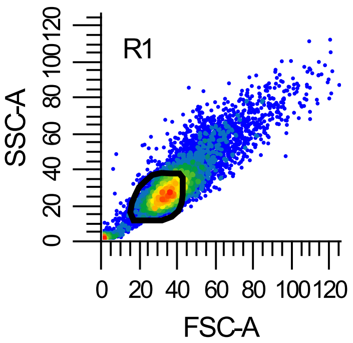

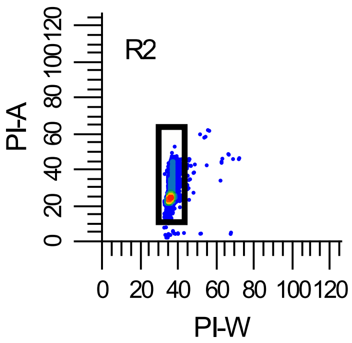

ModFit LT V4.1.7(Win)

FACSDiva Version 6.1.3

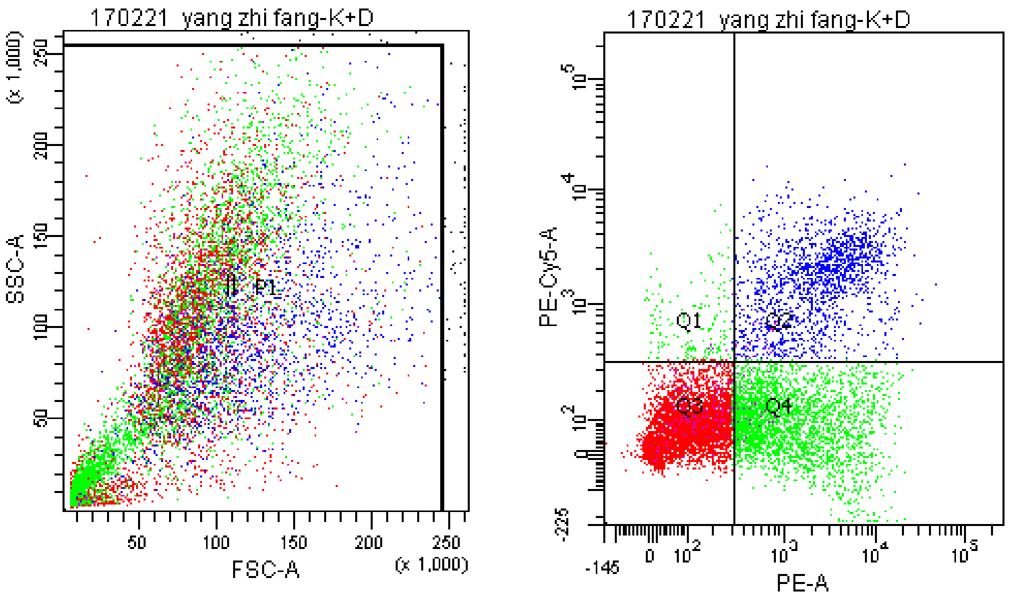

Tube: K+D

Population	#Events	%Parent	%Total
All Events	10,000	####	100.0
P1	9,841	98.4	98.4
Q1	178	1.8	1.8
Q2	1,709	17.4	17.1
Q3	4,828	49.1	48.3
Q4	3,126	31.8	31.3
P2	181	1.8	1.8

FACSDiva Version 6.1.3

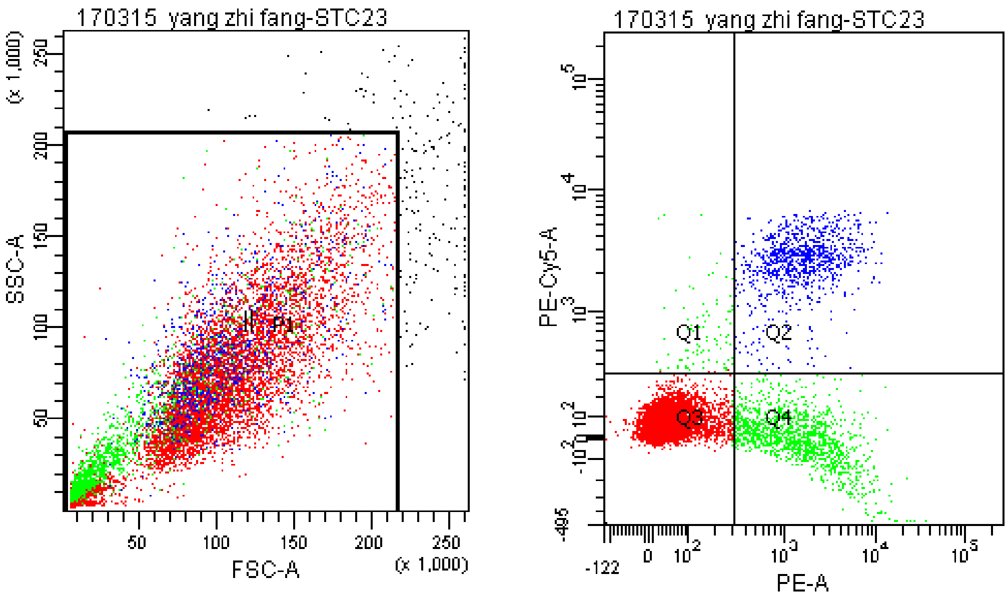

Tube: STC23

Population	#Events	%Parent	%Total
All Events	10,000	####	100.0
P1	9,764	97.6	97.6
Q1	86	0.9	0.9
Q2	950	9.7	9.5
Q3	7,429	76.1	74.3
Q4	1,299	13.3	13.0

FACSDiva Version 6.1.3

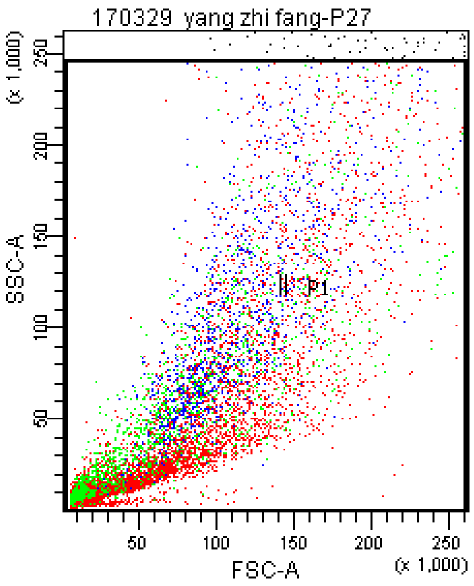

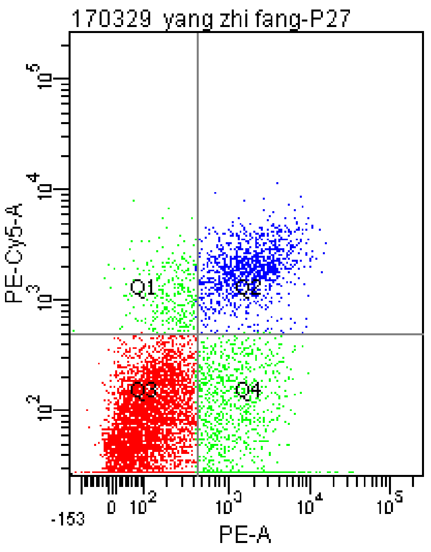

Tube: P27

Population	#Events	%Parent	%Total
All Events	10,000	####	100.0
P1	9,520	95.2	95.2
Q1	281	3.0	2.8
Q2	1,118	11.7	11.2
Q3	6,979	73.3	69.8
Q4	1,142	12.0	11.4

FACSDiva Version 6.1.3

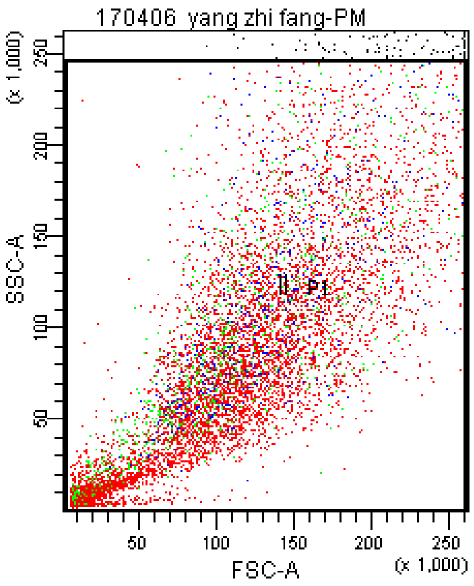

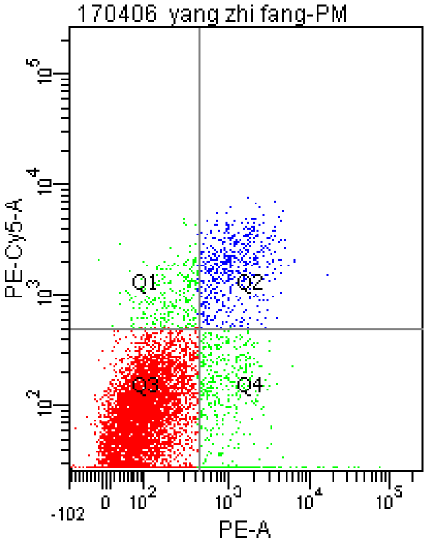

Tube: PM			
Population	#Events	%Parent	%Total
All Events	10,000	####	100.0
P1	8,853	88.5	88.5
Q1	248	2.8	2.5
Q2	541	6.1	5.4
Q3	7,505	84.8	75.0
Q4	559	6.3	5.6

FACSDiva Version 6.1.3

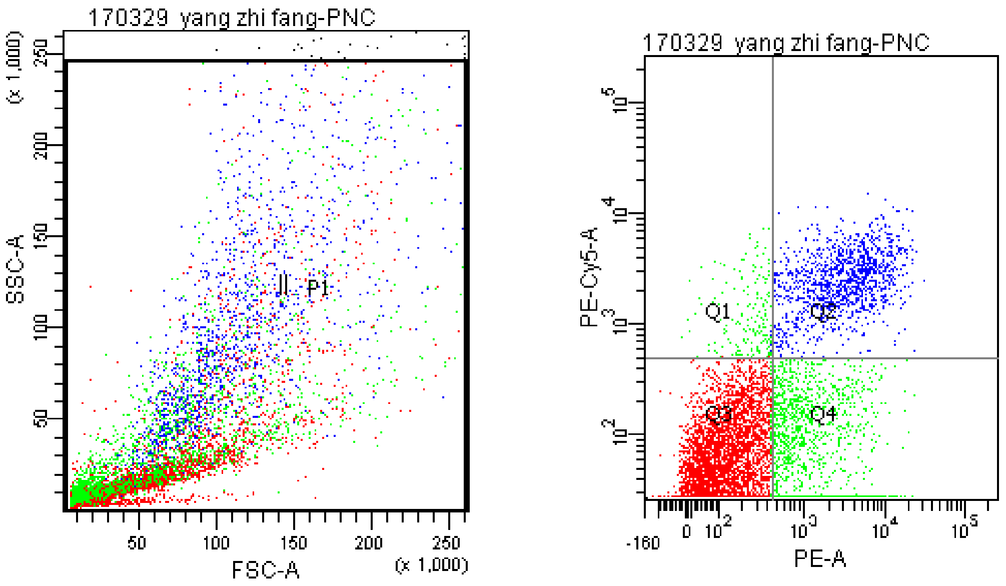

Tube: PNC

Population	#Events	%Parent	%Total
All Events	10,000	####	100.0
P1	9,744	97.4	97.4
Q1	174	1.8	1.7
Q2	1,233	12.7	12.3
Q3	6,750	69.3	67.5
Q4	1,587	16.3	15.9

FACSDiva Version 6.1.3

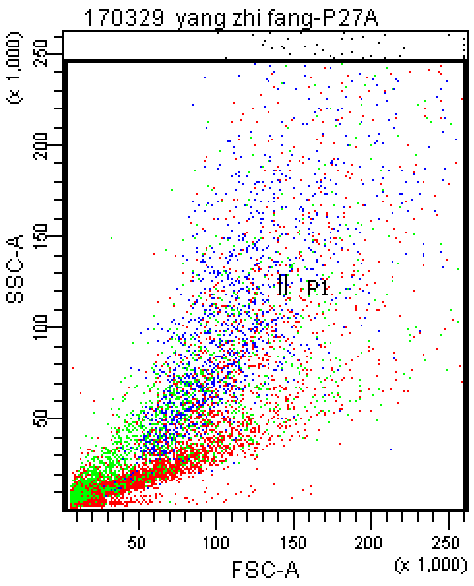

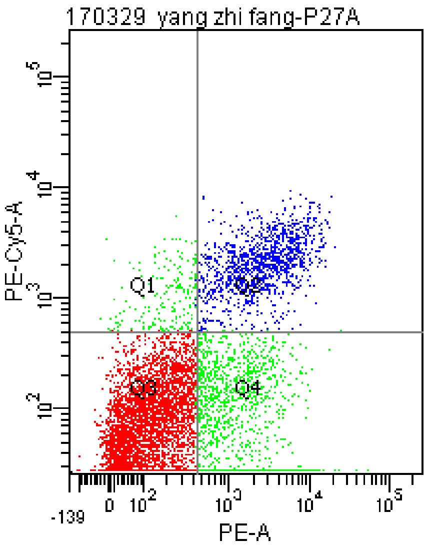

Tube: P27A

Population	#Events	%Parent	%Total
All Events	10,000	####	100.0
P1	9,677	96.8	96.8
Q1	191	2.0	1.9
Q2	1,275	13.2	12.8
Q3	6,822	70.5	68.2
Q4	1,389	14.4	13.9
